# Distribution of microbiota in cervical preneoplasia of racially disparate populations

**DOI:** 10.1186/s12885-022-10112-6

**Published:** 2022-10-18

**Authors:** Kunwar Somesh Vikramdeo, Shashi Anand, Jennifer Young Pierce, Ajay Pratap Singh, Seema Singh, Santanu Dasgupta

**Affiliations:** 1 Mitchell Cancer Institute, University of South Alabama, Mobile, AL 36604 USA; 2 Department of Pathology, College of Medicine, Mitchell Cancer Institute, University of South Alabama, 1660 Springhill Avenue, Mobile, AL 36604 USA; 3 Department of Biochemistry and Molecular Biology, University of South Alabama, Mobile, AL 36688 USA

**Keywords:** Cervix, Preneoplasia, Microbiome, HPV, Cancer risk

## Abstract

**Backgrounds:**

Microbiome dysbiosis is an important contributing factor in tumor development and thus may be a risk predictor for human malignancies. In the United States, women with Hispanic/Latina (HIS) and African American (AA) background have a higher incidence of cervical cancer and poorer outcomes than Caucasian American (CA) women.

**Methods:**

Here, we assessed the distribution pattern of microbiota in cervical intraepithelial neoplasia (CIN) lesions obtained from HIS (*n* = 12), AA (*n* = 12), and CA (*n* = 12) women, who were screened for CC risk assessment. We employed a 16S rRNA gene sequencing approach adapted from the NIH-Human Microbiome Project to identify the microbial niche in all CIN lesions (*n* = 36).

**Results:**

We detected an appreciably decreased abundance of beneficial *Lactobacillus* in the CIN lesions of the AA and HIS women compared to the CA women. Differential abundance of potentially pathogenic *Prevotella, Delftia, Gardnerella,* and *Fastidiosipila* was also evident among the various racial groups. An increased abundance of *Micrococcus* was also evident in AA and HIS women compared to the CA women. The detection level of Rhizobium was higher among the AA ad CA women compared to the HIS women. In addition to the top 10 microbes, a unique niche of 27 microbes was identified exclusively in women with a histopathological diagnosis of CIN. Among these microbes, a group of 8 microbiota; *Rubellimicrobium, Podobacter, Brevibacterium, Paracoccus, Atopobium, Brevundimonous, Comamonous, and Novospingobium* was detected only in the CIN lesions obtained from AA and CA women.

**Conclusions:**

Microbial dysbiosis in the cervical epithelium represented by an increased ratio of potentially pathogenic to beneficial microbes may be associated with increased CC risk disparities. Developing a race-specific reliable panel of microbial markers could be beneficial for CC risk assessment, disease prevention, and/or therapeutic guidance.

## Background

In the current setting, high-risk human papillomavirus (hrHPV) detection, Papanicolaou test (Pap test), and colposcopy-based screening have considerably reduced the overall rate of cervical cancer (CC) mortality in the USA [1]. However, significant racial health disparities exist in CC outcomes, posing a challenge to effective disease management [2–5]. Among various races, Hispanic/Latina (HIS) women have the highest rate of CC incidence and advanced staged diagnosis compared to other racial groups. The HIS women also have a higher mortality rate (9.5/100,000) compared to non-HIS women (7.5/100,000). Notably, the mortality rate among the HIS women is also slightly higher compared to the African American (AA) women [6]. On the other hand, CC associated mortality rate is double in AA women compared to Caucasian American (CA) women [7]. In terms of frequency, CC incidence is higher among AA compared to CA women (9/100,000 vs. 7.2/100,000). Similar to the HIS women, AA women have a higher incidence (60%) of CC, with an increased risk of advanced stage diagnosis compared to CA women.

Microbiota comprises a diverse population of bacteria, fungi, and viruses. It is an integral component of the human body, participating in various regulatory processes, including immune system function and metabolism. Loss of the beneficial components of the diverse microbiota and concomitant increase in their pathogenic counterpart may lead to chronic inflammation and transformation of the epithelial cells. The gastric epithelial cell resident *Helicobacter pylori* is an excellent example, whose increased accumulation is associated with a higher rate of gastric cancer [8, 9]. Diverse microbiota populations reside in the cervicovaginal canal of the female reproductive system. Changes in their composition may elicit inflammatory changes in the cervicovaginal epithelium, leading to an increased risk of cervical cancer. Notably, the cervicovaginal microbiota compositions may alter differentially in women of different racial backgrounds, posing a variable risk of CC development. In the present study, we examined the resident microbial compositions of the cervical intraepithelial lesions obtained from CA, AA, and HIS women, who were initially screened for CC risk assessment. We observed a lower abundance of the beneficial *Lactobacillus* and a concomitant increase in potentially pathogenic *Prevotella* and *Leucobacter* in AA and HIS groups compared to the CA women. A unique signature of twenty-seven microbes was identified exclusively in women with variable degrees of preneoplastic changes in their cervical epithelium (cervical intraepithelial neoplasia, grade 1–3), of which eight genera were detected only in the preneoplastic lesions obtained from the AA and HIS women.

## Methods

### Human samples and ethical statement

Cervical intraepithelial neoplasia (CIN) tissues from various grades (CIN1**-**CIN3) were collected from 36 women. Twelve samples each were obtained from CA, AA, and HIS women. (Table 1). All archived biospecimens were collected from the de-identified subject under an IRB-approved protocol from the University of South Alabama (#20–222). This study was approved by the Ethics Committee of Medicine, University of South Alabama. Informed consent was obtained from all the de-identified subjects, and only relevant clinical information such as age, grade, stage, diagnosis, HPV status, race, etc., was collected for statistical comparison. All methods were performed following the relevant guidelines and regulations.

**Table 1:** Demographic information of the women detected with cervical intraepithelial neoplasia (CIN) from various races. The cervical intraepithelial neoplastic lesions from all these women were sequenced for detecting various microbiomes

**Case #**	**Diagnosis**	**HPV**^1^ **status**	**Age**
HIS1^2^	CIN1^3^	Positive	26
HIS2	NIL^4^	Negative	46
HIS3	NIL	Negative	45
HIS4	CIN1	Negative	37
HIS5	CIN2	Positive	25
HIS6	CIN2/CIN3	Positive	20
HIS7	CIN2/CIN3	Positive	42
HIS8	CIN1	Negative	44
HIS9	CIN1	Positive	34
HIS10	CIN2	Positive	21
HIS11	CIN2	Positive	27
HIS12	CIN1	Negative	28
			**Mean = 32.92 ± 9.62**
AA1^5^	CIN3	None^6^	32
AA2	CIN3	Negative	31
AA3	CIN2/CIN3	Positive	40
AA4	NIL	Negative	26
AA5	CIN1	Positive	39
AA6	CIN1	Negative	25
AA7	NIL	Negative	42
AA8	CIN3	Negative	44
AA9	NIL	Positive	33
AA10	CIN2	Positive	41
AA11	NIL	Positive	47
AA12	CIN1	Positive	23
			**Mean = 35.25 ± 8.00**
CA1^7^	CIN2/CIN3	Positive	54
CA2	CIN3	Positive	51
CA3	CIN3	Positive	47
CA4	NIL	Positive	61
CA5	CIN2/CIN3	Positive	25
CA6	CIN3	None	41
CA7	NIL	Negative	62
CA8	CIN3	None	24
CA9	CIN2	Positive	38
CA10	CIN2	None	45
CA11	CIN2/CIN3	Positive	43
CA12	CIN2/CIN3	Negative	47
			**Mean = 44.42 ± 12.32**

^1 ^Human papilloma virus

^2 ^Hispanic

^3 ^cervical intraepithelial neoplasia

^4 ^negative for cervical intraepithelial neoplasia

^5 ^African American

^6 ^no information on HPV status

^7 ^Caucasian American

### 16S rRNA gene sequencing for microbiome detection

Cervical intraepithelial neoplasia lesions from AA (*n* = 12), CA (*n* = 12), and HIS (*n* = 12) subjects were processed (*n* = 36) for the microbiome sequencing. The 16S rRNA gene sequencing methods were adapted from the NIH-Human Microbiome Project as described previously, and specific guidelines for sample processing and data analysis were followed [10–12]. Briefly, we isolated bacterial genomic DNA utilizing MO BIO PowerSoil DNA Isolation Kit (MO BIO Laboratories, (MoBIO PowerSoil v3.4). We then performed PCR amplification of the 16S rDNA V4 region (Illumina 16Sv4 v1.2) by PCR using a set of specific primers described earlier [13]. Sequencing was carried out on the MiSeq platform (Illumina MiSeq v2 2 × 250 v1.8) using the 2 × 250 bp paired-end protocol yielding pair-end reads that overlap almost completely. The primers used for the amplification contained adapters for MiSeq sequencing and single-end barcodes, allowing pooling and direct sequencing of the PCR products [14].

### Quality control and compositional analysis

The pipeline data for the 16S rRNA gene assembles phylogenetic, and alignment-based approaches to maximize the data resolution described [13]. Based on the unique molecular barcodes, The read pairs were de-multiplexed. The resulting reads were merged using USEARCH v7.0.1090, equipped with a de novo built-in chimera filter. All singleton operational taxonomic units (OTUs) were discarded as described previously [13, 15–17]. To remove sequencing errors, Contig filters were employed as appropriate. The 16S rRNA gene sequences were clustered into Operational Taxonomic Units (OTUs), which were mapped to the SILVA database at 97% similarities to obtain phylum to genus taxonomies. The mapping of the OTUs was done utilizing an optimized version of the SILVA database, which exclusively contains the 16S v4 region for determining taxonomies. For abundance recovery, de-multiplexed mapping of the reads to the UPARSE OTUs was performed.

### Data analysis

The distribution of the microbiome populations was assessed in alpha diversity (AD) and taxa abundances (TA) through heat maps and customizable box/hierarchical plots as applicable. As described, appropriate statistical annotations were added to infer significant correlations with the metadata outcomes [13]. Kruskal–Wallis and Mann–Whitney tests were conducted for all the statistical data analyses with an appropriate FDR application utilizing the Bonferroni correction method. All the samples with total read counts below the designated threshold levels were excluded from further analysis. We employed alpha diversity analysis to measure diversity within a sample. The different alpha diversity metrics use the counts (richness) and distribution (evenness) of the OTUs within a sample as the basic values for these calculations. All the sequencing data have been deposited in the NIH sequence read archive (SRA, # PRJNA824515).

## Results

### Cervical cancer screening survey of the study population

We have examined microbiome diversity in 36 women from various ethnic groups with a primary diagnosis of cervical intraepithelial neoplasia (Table 1). The mean age of the HIS subjects was 32.92 ± 9.62; 35.25 ± 8.00 for the AA and 44.42 ± 12.32 for the CA subjects**.** Of the 12 CA cases, seven were positive for hrHPV infection, two were negative, and no information was available for the remaining three cases. Only two CA subjects exhibited mild (CIN1) to moderate (CIN2) dysplasia in their cervical epithelium. Seven of the 12 HIS cases were positive for hrHPV infection, and five were negative. Only two HIS subjects exhibited mild (CIN1) to moderate (CIN2) dysplasia in their cervical epithelium. Of the 12 AA cases, six were positive for hrHPV infection, five were hrHPV negative, and no information was available for one subject. All but four AA subjects exhibited mild (CIN1) to moderate (CIN2) dysplasia in the cervical epithelium.

### Overall sequence reads in the samples

Thirty-six biopsied samples from the uterine cervix were extracted and processed for 16Sv3-4 amplification and sequencing to determine microbiome profiles. All samples were included in the final analysis. A total of 251,993 sequencing reads were obtained from the 36 samples. Of these, 92.1% completed the merging and the quality filtering steps, and 1.36% of the resulting filtered reads mapped to the SILVA database after a QC step to remove potential contaminants.

### Nature and distribution of the microbiota in racially disparate population

**Fig. 1:**
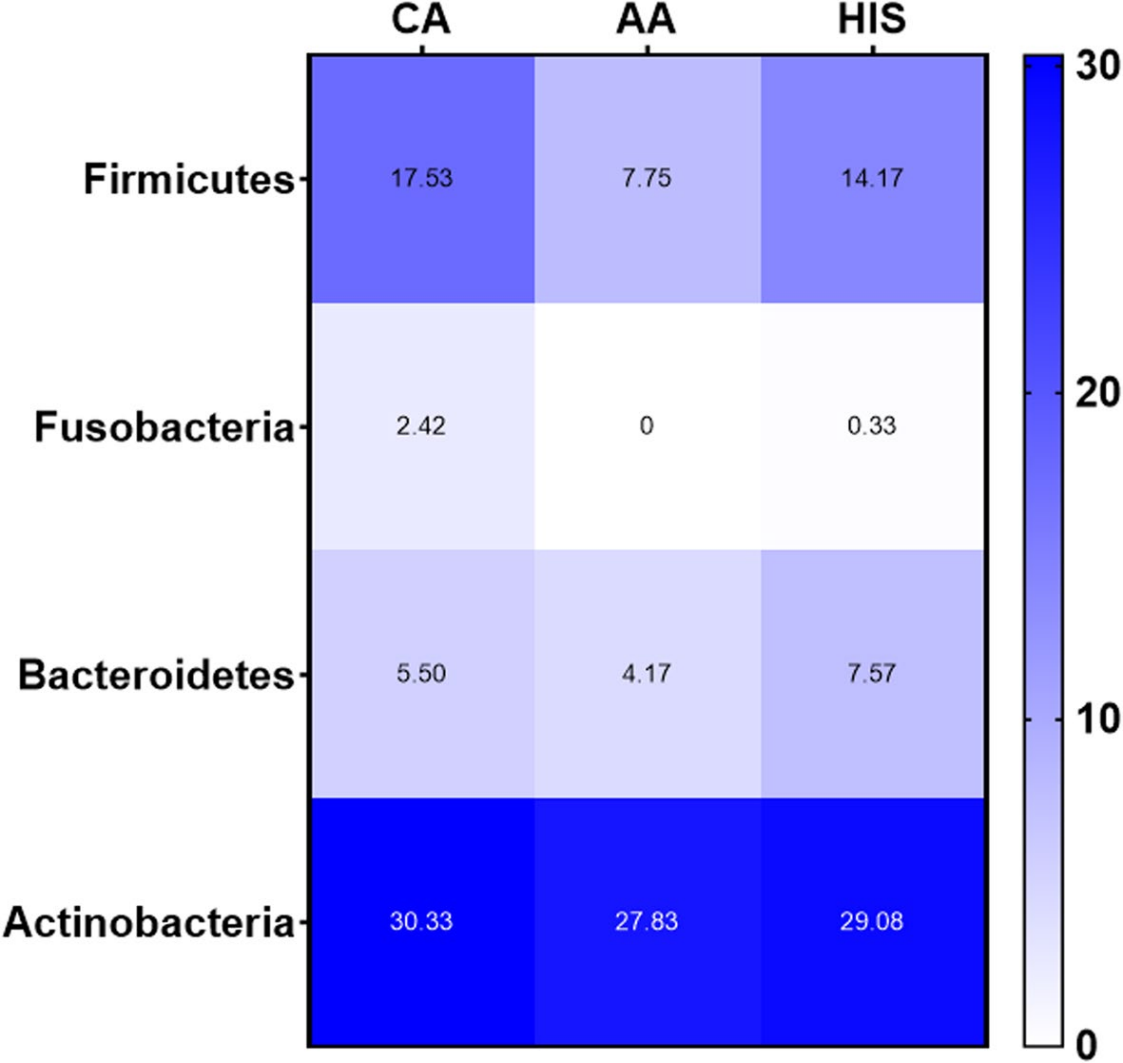
Major microbial Phyla were identified in women with various racial backgrounds. The differential level of abundance was presented as the mean abundance of the operational taxonomical unit. The mean abundance of the operational taxonomical unit was presented in each box in the various racial groups. A minimum average of 0.05% was considered for calculating the overall abundance. AA: African American; CA: Caucasian American; HIS: Hispanic/Latino

**Fig. 2:**
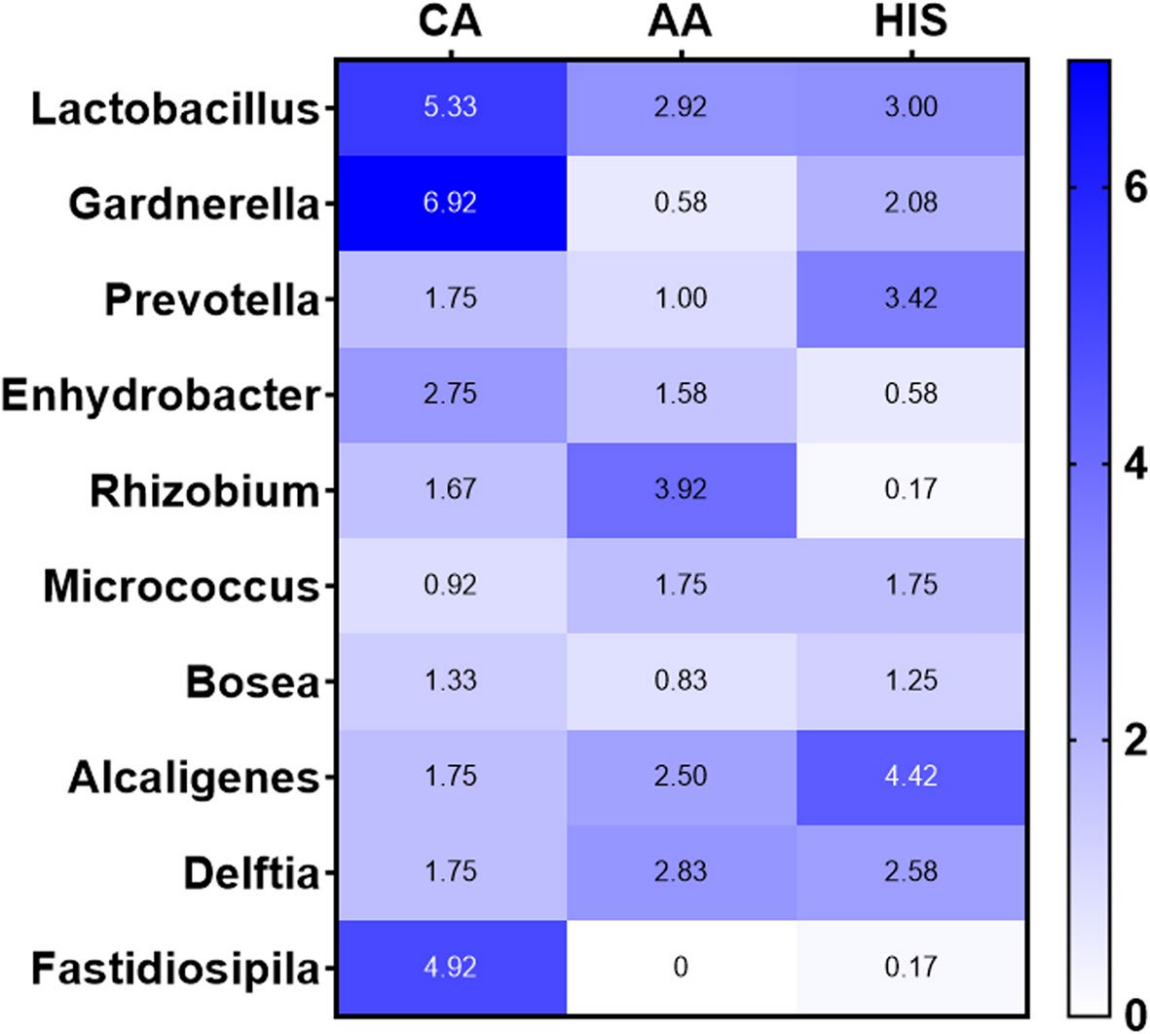
Differential abundance of the top 10 microbial genera identified in various racial groups. The differential level of abundance was presented as the mean of the operational taxonomical unit. The mean abundance of the operational taxonomical unit was presented in each box in the various racial groups. A minimum average of 0.05% was considered for calculating the overall abundance. AA: African American; CA: Caucasian American; HIS: Hispanic/Latino

**Fig. 3:**
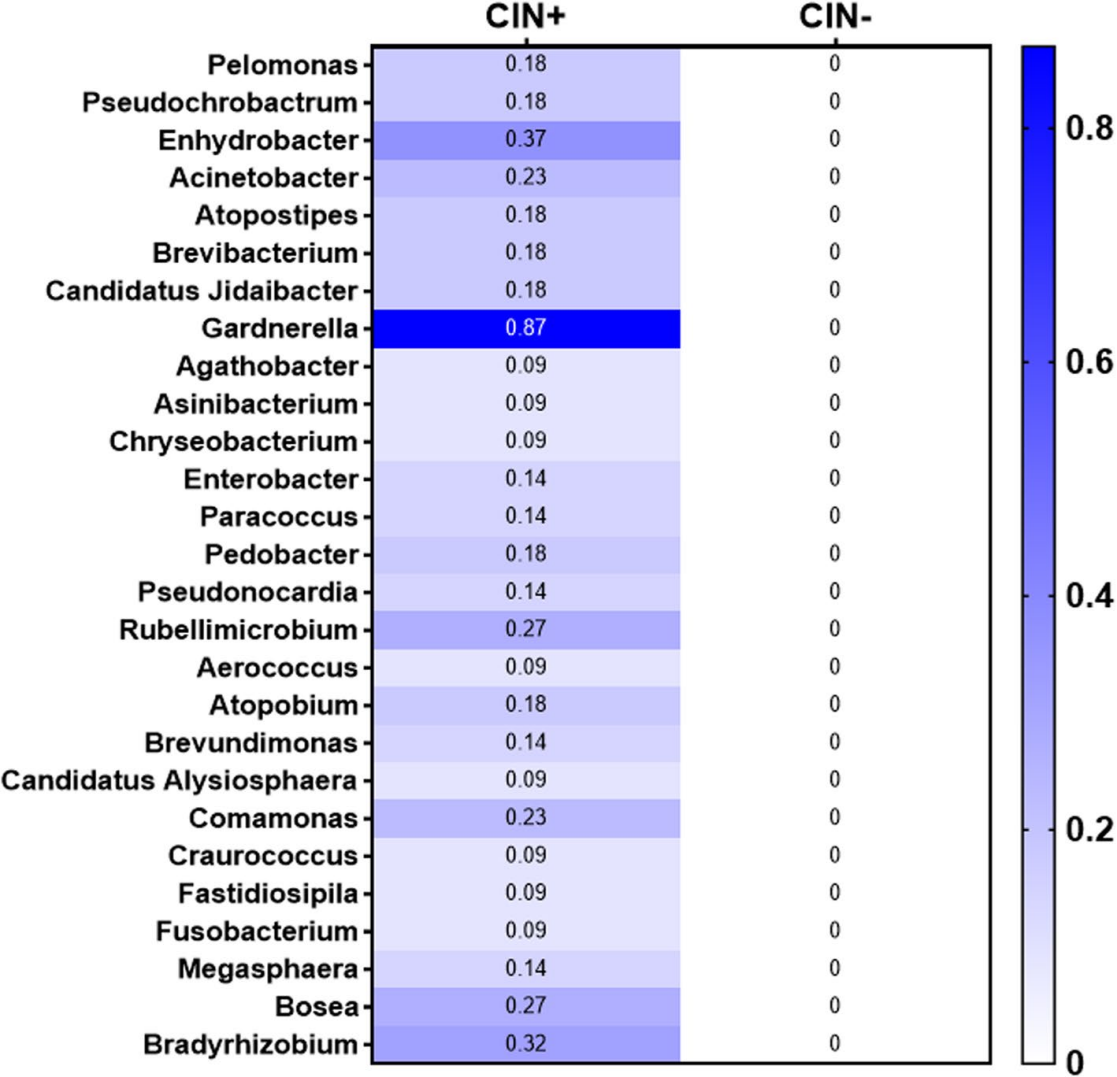
Microbial genera were identified exclusively in the cervical intraepithelial neoplasia (CIN) lesions from African American (AA) and Hispanic/Latina (HIS) women. The differential level of abundance was presented as the mean of the operational taxonomical unit. A minimum average of 0.05% was considered for calculating the overall abundance. CA: Caucasian American

**Fig. 4:**
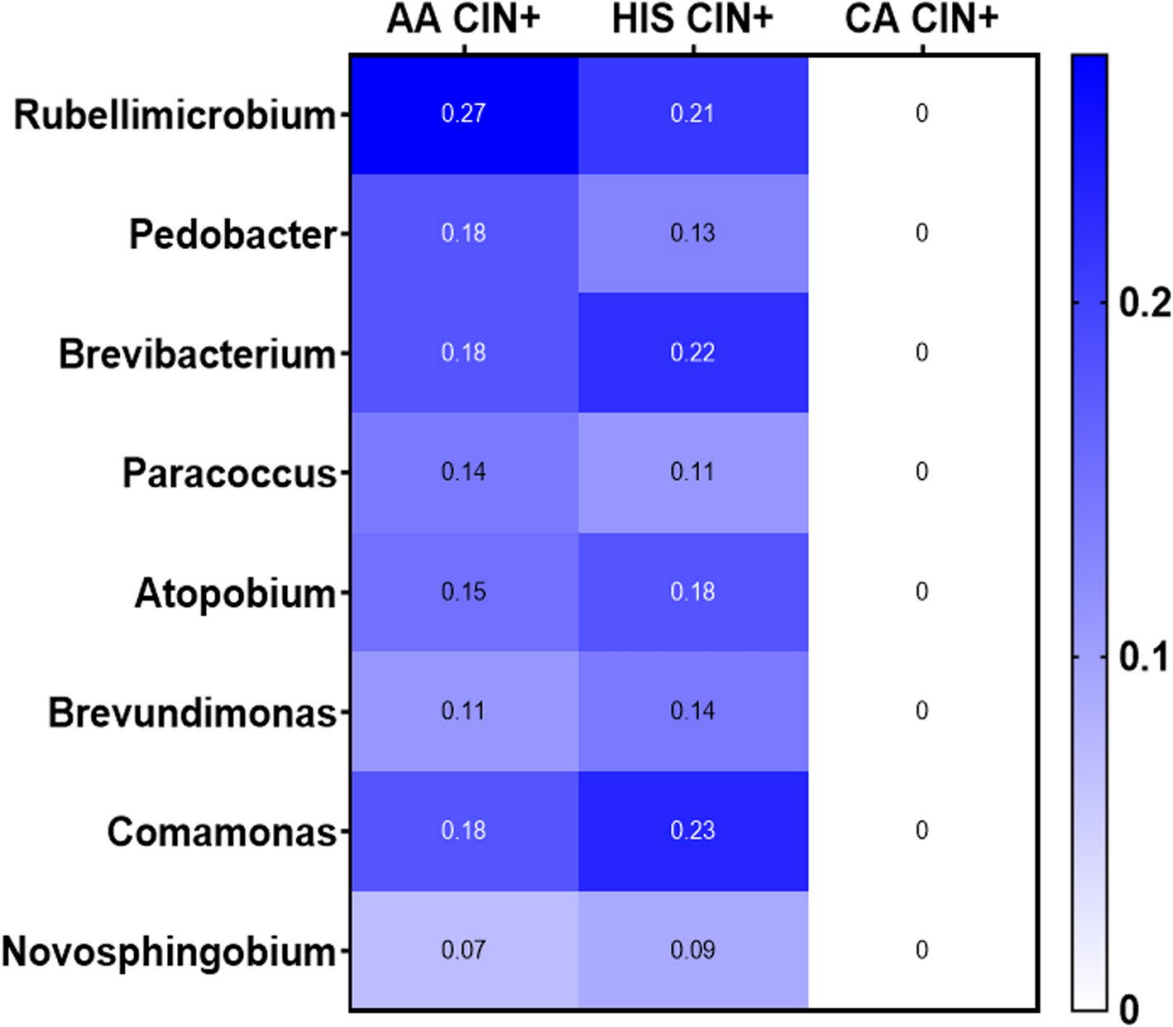
Microbes detected only in the cervical intraepithelial neoplasia (CIN) lesion (lower abundance) from African American (AA) and Hispanic/Latina (HIS) women. The differential level of abundance was presented as the mean of the operational taxonomical unit. The mean abundance of the operational taxonomical unit was presented in each box in the various racial groups. A minimum average of 0.05% was considered for calculating the overall abundance. CA: Caucasian American

**Fig. 5:**
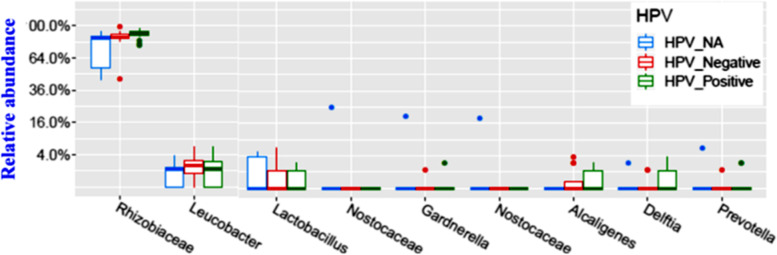
The pattern of distribution of the operational taxonomic units (OTUs) in the cervical intraepithelial neoplasia lesions across HPV positive and negative women with diverse racial backgrounds. A minimum average of 0.05% was considered for calculating the overall abundance. HPV-NA: No information on HPV status

**Fig. 6:**
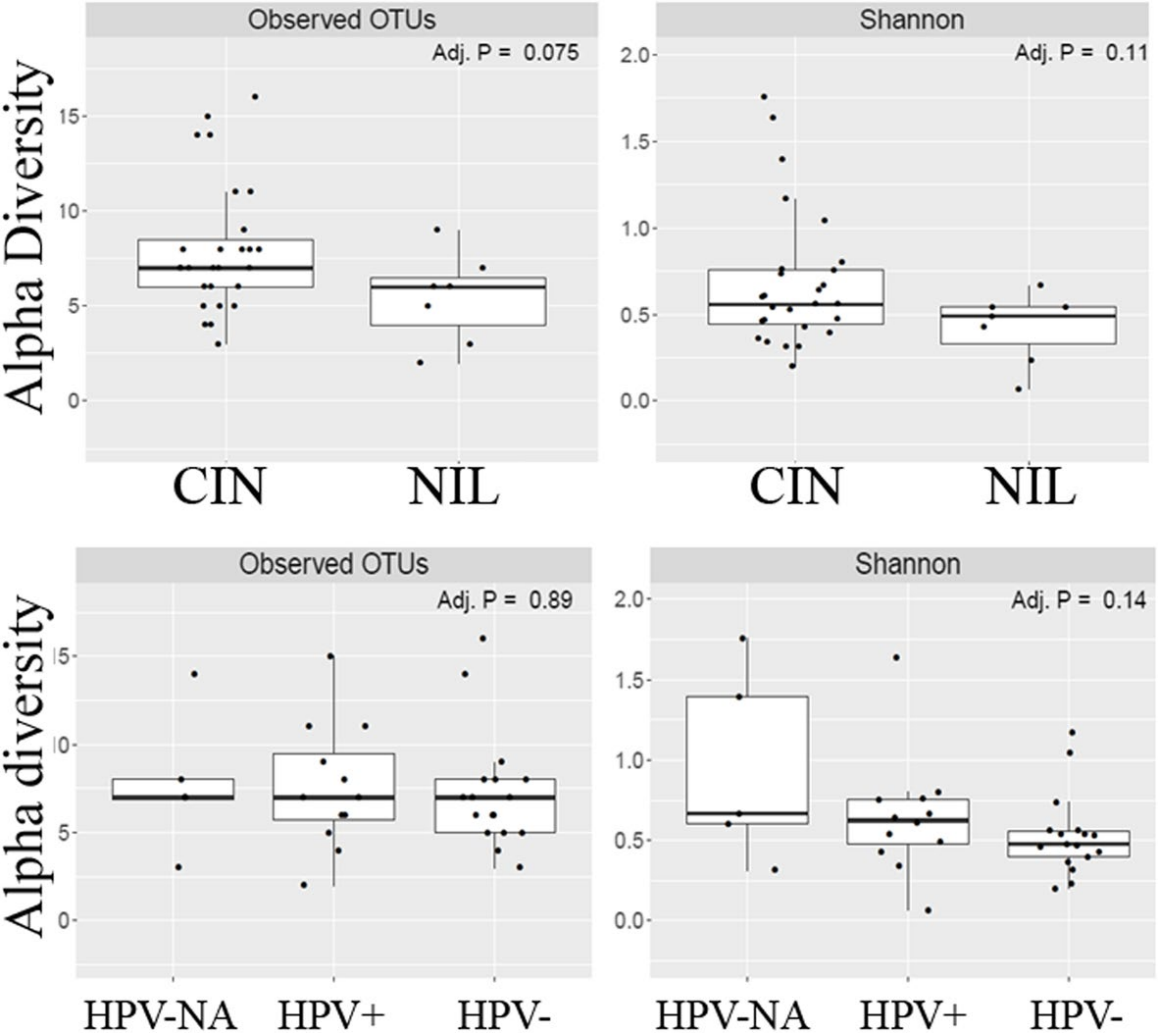
Ordination plots for alpha diversity to measure intra-sampler diversity. For these calculations, the alpha diversity metrics use both counts (richness) and distribution (evenness, Shannon Index) of the operational taxonomic units (OTUs) within a sample as the basic values. A minimum average of 0.05% was considered for calculating the overall abundance in all cases. CIN: cervical intraepithelial neoplasia; NIL: No detection of cervical intraepithelial neoplasia; HPV-NA: no information on HPV status; HPV + : women positive for HPV infection; HPV-: women negative for HPV infection

Two hundred fifty-nine unique Operational Taxonomic Units (OTUs) were observed in the samples analyzed. The average and median number of OTUs per sample were 15 and 13, respectively. The OTUs were classified across 13 different phyla, 74 families, and 142 genera. Overall, in all three racial groups, a considerable abundance of microbiota was evident from 4 major phyla, including, Firmicutes, Fusobacteria, Bacteroidetes, and Actinobacteria (Fig. 1). Within these 4 phyla, we have identified a considerable and differential abundance of 10 genera in the various racial groups, including *Lactobacillus, Gardnerella, Prevotella, Enhydrobacter, Rizhobium, Micrococcus, Alcaligenes, Delftia, Fastidiosipila and Bosea* (Fig. 2). Notably, among these 10 genera, a lower abundance (twofold) of *Lactobacillus, a common cervicovaginal microbe,* was noted in both AA and HIS women compared to the CA women. On the other hand, a higher abundance (1.5–fourfold) of *Gardnerella, Enhydrobacter, Bosea, Delftia, and Fastidiosipila* was also evident in the CA group compared to the AA and HIS women. Of note, we observed a 2–threefold higher abundance of *Prevotella* in HIS women compared to CA and AA women. Considering the variable degree of abundance (high-low), we have identified a panel of 27 microbes, which are exclusively present in women with a primary diagnosis of CIN but not in women with histologically normal-appearing cervical epithelium (Fig. 3). In addition to the highly abundant 10 genera detected in various racial populations (Fig. 2), a comparatively lower abundance of a panel of 8 microbes; *Rubellimicrobium, Podobacter, Brevibacterium, Paracoccus, Atopobium, Brevundimonous, Comamonous, and Novospingobium* was detected exclusively in CIN lesions from AA and HIS subjects (Fig. 4). We also identified a differential abundance of a group of 9 microbes in HPV-positive and HPV-negative CIN lesions from various racial groups (Fig. 5). We determined alpha diversity for species richness and the evenness between various groups considering CIN and HPV positivity. However, we did not observe any significant difference in richness or evenness (Fig. 6).

## Discussion

Microbiota represents a collective composition of bacteria, viruses, and fungi residing in the human body that participate in various metabolic processes [18–20]. An imbalance, commonly known as dysbiosis in the microbiota through an increased accumulation of pathogenic microbes and/or a decrease in the beneficial ones may initiate several pathobiological changes, including genomic instability, cellular proliferation, chronic inflammation, metabolic reprogramming, and immunosuppression leading to various diseases, including cancer [21–24].

Despite appreciable advancement in disease prevention strategies, women of diverse racial backgrounds carry a disproportionate burden of CC, warranting a deeper understanding of the underlying contributing factors, followed by refinement of current risk assessment strategies. Cervical carcinomas often develop through precursor cervical intraepithelial neoplastic lesions and are primarily driven by HPV oncogenesis [25, 26]. Interestingly, recent studies suggest that altered residential patterns of the microbiota in the cervicovaginal environment could be associated with the development of various diseases, including malignant transformation [27]. However, the compendium of microbiota adherent during early transformative changes of the cervical epithelium in populations with variable CC risk remains less well explored. Our pilot study identified the diverse microbiome in cervical intraepithelial neoplasia specimens obtained from HIS, AA, and CA women. It determined their correlation with preneoplastic changes and HPV status in various racial groups. This is the first study undertaking a comparative analysis of microbiota in CA, AA, and HIS women with a primary diagnosis of cervical intraepithelial neoplasia.

The most abundantly found and the beneficial resident microbe in the female genital tract is *Lactobacillus* [28]. *Lactobacillus species* appear to exert their protective function by metabolizing available glycogen from the vaginal epithelial cells undergoing periodic changes. The metabolization of glycogen by *Lactobacillus* creates a low pH environment, which aids in preventing the accumulation and adherence of pathogenic microbes therein [29]. Moreover, *Lactobacillus* is one of the microbes capable of altering the host’s microbiome, improving the immune response, reducing inflammation, and promoting HPV clearance [30]. Detection of the decreased abundance of *Lactobacillus* in the HIS and AA women compared to the CA women suggests an increased risk of colonization of the pathogenic microbes facilitating immunosuppression and subsequent HPV-mediated cervical carcinogenesis.

This notion is further supported by detecting a higher abundance of CC-associated pathogenic microbe *Prevotella* in HIS women [31]. Notably, *Prevotella* is a unique microbe harboring estrobolome with β-glucaronidase and/or β-galactosidase activity, which is known to promote breast tumorigenesis [13]. Considering the CC facilitating role of estrogen [32, 33]. *Prevotella* may contribute to augmenting estrogen metabolism through its estrobolome activity. Along with *Prevotella*, *Gardnerella* and *Fastidiosipila* are emerging as potential risk factors and/or biomarkers for developing moderate CC development [34, 35]. Thus, an increased abundance of *Gardnerella* and *Fastidiosipila,* as identified in our study in racially disparate populations, may potentially be associated with the development of an immunosuppressive environment, thereby increasing the risk of HPV-mediated oncogenic transformation of the cervical epithelium. In a recent study, *Delftia* has been suggested as a microbiological hallmark of cervical preneoplasia due to its remarkable enrichment in both low and high-grade CIN lesions [36]. Detection of an increased abundance of *Delftia* in CIN lesions obtained from AA and HIS women further supports this notion.

Detection of a unique niche of a potentially pathogenic group of microbiota, exclusively in women with CIN lesions, further suggests that these microbiomes may play a critical role in cervical epithelial cells’ transformation alone and/or in concert with hrHPV integration. In addition, the detection of certain pathogenic microbes exclusively in AA and HIS women indicates the adherence to distinct microbiota niches in these women, which could be associated with disparate risks of CC development. Detection of *Atopobium* was reported in CIN2/3 lesions [37]. In this context, exclusive detection of Atopobium in CIN lesions from AA and HIS women suggests their role in transforming the normal cervical epithelium. Similarly, *Brevibacterium* and *Novosphingobium* were detected in CIN lesions from Mexican women [38], which were also seen exclusively in the AA women derived CIN lesions and HIS women in our study. Findings from this pilot study rationally instigate the development of a consensus panel of altered microbiome signatures through a comprehensive analysis of a large number of samples, which is our immediate goal. In the longer run, in concert with the standard risk assessment practice [39], routine screening of racially disparate populations for “microbiome dysbiosis” using a pre-defined marker panel may be helpful to improve the current disease prevention strategies further and reduce the disparity gaps.

In summary, we detected a differential abundance of a group of microbiota in various racial groups of women with a primary diagnosis of cervical preneoplasia, which may be associated with disparate CC risk outcomes and subsequent development and progression. The development of a race-specific reliable panel of microbial markers could be beneficial for better CC risk stratification and disease prevention and/or therapeutic guidance.

## Data Availability

All the sequencing data have been deposited in the NIH sequence read archive (SRA, # PRJNA824515). This Sequence Read Archive (SRA) submission has been released on 2022–08-25 and is available at https://submit.ncbi.nlm.nih.gov/subs/bioproject/SUB11231366/overview
